# Saunders’ Medical Hand-Atlases

**Published:** 1903-02

**Authors:** 


Saunders' Medical Hand-Atlases.—Atlas and Epitome of Abdominal Hernias. By Dr. George Sultan, First Assistant in the Surgical Clinic in Göttingen, Prussia. Authorized translation from the German. Edited by William B. Coley, M. D., Clinical

Lecturer on Surgery, Columbia University; Surgeon to the General Memorial Hospital; Assistant Surgeon to the Hospital for Ruptured and Crippled, New York City. With 119 illustrations, 36 of them in colors, 280 pages. W. B. Saunders & Co., Philadelphia and London. 1902. Price, $3.00, net.

An accurate knowledge of the operative treatment of hernias is as essential to the general practitioner as to the surgeon. Acute strangulation is of comparatively frequent occurrence and always demands immediate energetic surgical intervention, therefore every practitioner ought to be prepared to deal with such an emergency.

This atlas with its comprehensive text and its numerous superbly executed illustrations certainly excels any other work in English dealing with the operative aspect of the subject. R.

